# Association between substance use and psychosocial characteristics among adolescents of the Seychelles

**DOI:** 10.1186/1471-2431-11-85

**Published:** 2011-10-11

**Authors:** Heba Alwan, Bharathi Viswanathan, Valentin Rousson, Fred Paccaud, Pascal Bovet

**Affiliations:** 1 Institute of Social and Preventive Medicine (IUMSP), University Hospital Center and University of Lausanne, Lausanne, Switzerland; 2 Ministry of Health, Section of Non Communicable Diseases, Victoria, Republic of Seychelles

## Abstract

**Background:**

We examined the associations between substance use (cigarette smoking, alcohol drinking, and cannabis use) and psychosocial characteristics at the individual and family levels among adolescents of the Seychelles, a rapidly developing small island state in the African region.

**Methods:**

A school survey was conducted in a representative sample of 1432 students aged 11-17 years from all secondary schools. Data came from a self-administered anonymous questionnaire conducted along a standard methodology (Global School-based Health Survey, GSHS). Risk behaviors and psychosocial characteristics were dichotomized. Association analyses were adjusted for a possible classroom effect.

**Results:**

The prevalence of cigarette smoking, alcohol drinking and cannabis use was higher in boys than in girls and increased with age. Age-adjusted and multivariate analyses showed that several individual level characteristics (e.g. suicidal ideation and truancy) and family level characteristics (e.g. poor parental monitoring) were associated with substance use among students.

**Conclusions:**

Our results suggest that health promotion programs should simultaneously address multiple risk behaviors and take into account a wide range of psychosocial characteristics of the students at the individual and family levels.

## Background

Substance use among adolescents can lead to a variety of detrimental consequences. Cigarette smoking, alcohol drinking and cannabis use can increase accidental or intentional injuries, commission of crimes, mood disorders, and mortality, and can complicate normal psychosocial development [1,2].

Adolescents are at risk for engaging in several risk behaviors as they are continuously exposed to a number of inducing influences, e.g., through mass media, publicity, video clips, celebrity media reports, and peer pressure [3-5]. Furthermore, many adolescents engage in substance use for experimental purposes [6-8]. A resiliency approach provides a framework for understanding why some youth do not engage in these behaviors [4,9]. Parental support [10], parent-child communication [11] and parental monitoring [12] are examples of protective characteristics that reinforce adolescent resiliency.

On the other hand, the risk factor approach aims to identify and subsequently prevent, reduce or eliminate precursors of risk behaviors [2]. Risk factors of substance use by children typically include substance use in the family or by peers [13], poor family management [12,14], deliquescent family structure [12], poor physical and mental health [4,8,15], peer crowd affiliation [16], poverty, and racial stigma [2,17]. The role of the family in predicting adoption of risk behaviors by youth has been emphasized, including poor parental monitoring and support and poor parent-child communication [12,14].

The degree of risk taking by adolescents can be viewed as the result of a complex interaction between risk factors and protective factors [17]. For example, a child from a single-parent family (risk factor) is less likely to engage in risk behaviors in the presence of good parenting (protective factor) [12]. This emphasizes the need to study risk behaviors in youth using a comprehensive approach that takes into account both protective characteristics and risk factors.

Assessing substance use in the Seychelles is useful in view of the scarce data in the African region [18-21]. While substance use has been extensively researched in youth in Western countries [1,4,8,11-14,16,22-27], socio-cultural differences between Western and African countries may underlie differences in the prevalence and correlates of risk behaviors across regions [18-21]. Existing data indicate that substance use poses a significant problem among adolescents in the Seychelles. For example, around 26% of school-aged children in the Seychelles have smoked during the past 30 days [28] as compared to 14-19% of adolescents in the United States [19,23]. Moreover, the prevalence of smoking and drinking among youth is fairly high in Seychelles compared to several other African countries [29], perhaps partly due to the relatively higher purchasing power in the former than the latter, and fairly high social tolerance to drinking in Seychelles [30]. In this study, we examined the association between substance use (tobacco, alcohol, and cannabis use) and protective characteristics and risk factors among adolescents in the Seychelles.

## Methods

The data in this study come from the Global School-based Health Survey (GSHS), which was conducted for the first time in the Seychelles in 2007 [31]. The GSHS, a school-based survey developed by the World Health Organization, the Centers for Disease Control and Prevention (Atlanta) and other international agencies, aims to provide a common methodology for collecting data on a broad set of risk behaviors and psychosocial characteristics among students worldwide (http://www.cdc.gov/gshs/questionnaire/index.htm). Consistent with the GSHS methodology, a two-stage cluster sample design was used to produce a representative sample of all students in grades S1-S4 in all public and private schools in Seychelles (school is compulsory up to the S4 level in the Seychelles, which correspond to the 10^th ^year of school after crèche). The first-stage sampling frame consisted of all schools containing the grades S1, S2, S3, and S4. Schools were selected with probability proportional to school enrolment size. In Seychelles, all the 13 schools containing S1-S4 classes in the Seychelles were selected in the study. The second-stage sampling frame consisted of an equal-probability sampling (with a random start) of all S1-S4 classes in the selected (13) schools: 64 classrooms were selected from a total of 274. All students in the sampled classrooms were eligible to participate in the survey. In total, 1432 students from the selected 64 classrooms from the 13 schools participated in the survey, corresponding to a participation rate of 82%.

The survey took place on the same day in all selected classes. The distribution of questionnaires and collection of the answer sheets involved 33 survey officers, with one officer assigned to each participating class. Survey officers had been trained during a one-day workshop. The students and parents were not informed prior to the survey in view of the absence of invasive investigations or physical measurements, the possibility for declining participation allowed to all students, and the anonymous nature of the questionnaire. The research committee of the Ministry of Health had approved the study including the fact that parental informed consent was not necessary. There were 35 participants aged 11 years (2%), 226 aged 12 (16%), 325 aged 13 (23%), 296 aged 14 (21%), 307 aged 15 (22%), 191 aged 16 (13%), and 47 aged 17 (3%).

Data were collected using a self-administered and anonymous questionnaire that was designed along the standard GSHS methodology. The GSHS core questionnaire includes 10 core questionnaire modules containing 3-7 questions as well as several additional country-specific questions. The questions chosen from the GSHS module must be used without modification (except for translation into the local language). In Seychelles, we used questions from the modules on tobacco, alcohol, drug use, mental health, and protective characteristics.

Different cutoffs have been applied in studies that assessed risk behaviors and associated characteristics among adolescents [4,22,23]. In this study, risk behaviors were dichotomized on the basis of definitions used by prior studies [4,22,23]. Smoking was defined as a response of "one or more days" to the question "During the past 30 days, on how many days did you smoke cigarettes?" Alcohol use was defined as a response of "one or more days" to the question "During the past 30 days, on how many days did you have at least one drink containing alcohol?" Cannabis use was defined as a response of "one or more times" to the question "During the past 30 days, how many times have you used drugs such as cannabis, marijuana or hashish (do not include heroin, cocaine, or ecstasy)". While it should be acknowledged that positive answers may also identify students who are only engaging in experimental "one-time" experiences, it is likely that the behaviors so defined most often reflect a more habitual behavior (hence a true "risk behavior" for the majority of students). Moreover, it should be noted that substantial proportions of adolescents progress to regular substance use after occasional use, a proportion that may range from 28% to 80% [27,32].

Four questions in the "GSHS mental health module" assess psychological characteristics: "During the past 12 months, how often have you felt lonely?", "During the past 12 months, did you ever feel so sad or hopeless almost every day for two weeks or more in a row that you stopped doing your usual activities?", "During the past 12 months, how often have you been so worried about something that you could not sleep at night" (referred to hereafter as "insomnia"), and "During the past 12 months, did you ever seriously consider attempting suicide?" Responses to these questions were on a 5-point Likert-type scale ranging from "never" to "always". Also part of the "GSHS mental health module", social interaction was assessed by asking "How many close friends do you have?" As there were only 5.8% of students who reported having no friends, the category for low social interaction was defined as having 0-1 friends (19.9% of all students).

We used the following three questions from the "GSHS protective factors module" to assess perceived parental monitoring and bonding: "During the past 30 days, how often did your parents or guardians check to see if your homework was done?"; "During the past 30 days, how often did your parents or guardians understand your problems and worries?"; and "During the past 30 days, how often did your parents or guardians really know what you were doing with your free time?" Responses to these questions were on a 5-point Likert-type scale ranging from "never" to "always". Questions on parenting practices, e.g. perceived parental monitoring or bonding, are commonly assessed among adolescents [11,33] as the relationships of adolescents with their parents, as perceived by the adolescents, may affect their behaviors [33]. Also part of this module, truancy was assessed by the question "During the past 30 days, on how many days did you miss school without permission?"

Finally, we added a question on pocket money available to the students ("How much pocket money do you get every day on average?") This variable has been employed in similar studies [16,21,25,34] to reflect, to a certain extent, the socioeconomic status of the participants' parents.

Differences in the prevalence of baseline characteristics between girls and boys were tested using the chi-squared test. We considered cigarette smoking, alcohol, and cannabis use (i.e. "risk behaviors") as the response variables. In all analyses, both the response variables and the explanatory variables were dichotomized. We used age-adjusted logistic regression to examine the associations between each of the three risk behaviors and the explanatory variables, separately for boys and girls. To account for a possible clustering effect, a random classroom effect has been included in all logistic regression models, using the xtlogit command of Stata 9.2. An estimate of the intraclass correlation is also provided, which is a measure of how closely students in a classroom resemble to each other regarding the outcome variables (technically, intraclass correlation is the percentage of the outcome variability that is due to the classroom effect from the variability that is not explained by the other factors in the model; this is being measured using an underlying latent continuous variable from which the outcome is a dichotomization) [35]. Multivariate analysis including several psychosocial variables has also been performed. Since several characteristics in the GSHS represent same constructs (e.g. several questions on parental control, etc), we used backward stepwise regression analysis to obtain parsimonious models. Age was included in all models. All participants were included in the analyses with the exception of 15 students in whom data regarding gender were missing (n = 1417). Analyses were performed using Stata 9.2 and p values < 0.05 were considered significant.

## Results

The prevalence of the variables under study appears in Table 1. Mean age for both girls and boys was 14 years (SD 1.4). During the 30 days prior to the study, 22% of boys vs. 11% of girls had smoked at least one cigarette (p < 0.001), 60% of boys vs. 56% of girls had consumed one alcoholic drink (ns), and 19% of boys vs. 4% of girls had used cannabis at least once (p < 0.001).

**Table 1:** Baseline characteristics among boys and girls aged 11-17 years

	Boys (n = 677)*		Girls (n = 740)*		
		
	%	95% CI	%	95% CI	*P*
Smoked ≥ 1 cigarette in past 30 days	22.0	18.7-25.3	10.6	8.3-12.9	0.000
Drank alcohol ≥ 1 times in past 30 days	59.7	55.6-63.9	56.2	52.3-60.0	ns
Used cannabis ≥ 1 time in past 30 days	19.2	16.1-22.2	3.7	2.3-5.1	0.000
Felt sad or hopeless ≥ 2 weeks in a row in past 12 months	27.7	24.2-31.2	35.0	31.5-38.5	0.004
Often felt lonely in past 12 months	10.4	8.1-12.8	15.2	12.6-17.7	0.009
Was so worried about something that could not sleep at night in past 12 months	10.0	7.7-12.2	12.1	9.7-14.5	ns
Seriously considered attempting suicide in past 12 months	15.9	13.1-18.7	18.4	15.6-21.2	ns
Parents rarely/never check homework	37.9	34.0-41.7	38.6	35.0-42.2	ns
Parents rarely/never understand problems	37.5	33.6-41.4	39.0	35.4-42.7	ns
Parents rarely/never know what child is doing	38.0	34.1-41.9	29.8	26.4-33.2	0.002
Pocket money ≥ 25 rupees per day	23.5	20.2-26.9	21.7	18.6-24.7	ns
School absence without permission ≥ 1 day in past 30 days	39.1	35.2-43.0	25.0	21.8-28.2	0.000
Has less than 2 close friends	19.1	16.0-22.1	21.9	18.9-25.0	ns

*Missing values vary from 8 to 252 according to the selected baseline characteristics.

Table 2 shows the age-adjusted associations between the three considered risk behaviors and each of the explanatory characteristics among boys. Hence, each line of the Table (explanatory characteristics) represents a separate (age-adjusted) model. The ranges of the sample sizes and intraclass correlations for the age-adjusted models are shown at the bottom of the table. Considering attempting suicide, insomnia, truancy, and parents who rarely/never know what their children are doing were positively associated with all risk behaviors (smoking, alcohol, and cannabis use). Students who often felt lonely and had parents who rarely/never check their homework were also more likely to report smoking. Boys who felt sad or lonely, who had less than two close friends, or whose parents rarely/never understood their problems were more likely to report cannabis use.

**Table 2:** Age-adjusted associations between risk behaviors and psychosocial characteristics among boys

	Smoking			Alcohol use			Cannabis use		
	
Variable	OR	95% CI	*P*	OR	95% CI	*P*	OR	95% CI	*P*
Felt sad or hopeless ≥ 2 weeks in a row in past 12 months	1.4	0.9-2.2	ns	1.2	0.8-1.8	ns	2.1	1.3-3.5	0.002
Often felt lonely in past 12 months	2.4	1.3-4.5	0.008	1.3	0.7-2.3	ns	2.9	1.6-5.5	0.001
Was so worried about something that could not sleep at night in past 12 months	2.8	1.5-5.2	0.002	2.1	1.0-4.3	0.039	4.9	2.7-9.1	0.000
Seriously considered attempting suicide in past 12 months	2.6	1.5-4.4	0.001	1.9	1.1-3.2	0.023	3.8	2.2-6.5	0.000
Parents rarely/never check homework	1.5	1.0-2.4	0.053	1.4	0.9-2.1	0.091	1.4	0.9-2.2	Ns
Parents rarely/never understand problems	1.3	0.9-2.1	ns	1.3	0.9-2.0	ns	1.7	1.1-2.7	0.027
Parents rarely/never know what child is doing	2.1	1.4-3.3	0.000	1.8	1.2-2.7	0.002	1.8	1.2-2.9	0.010
Pocket money ≥ 25 rupees per day	1.4	0.8-2.2	ns	1.2	0.8-2.0	ns	0.8	0.5-1.6	Ns
School absence without permission ≥ 1 day in past 30 days	3.9	2.5-6.1	0.000	2.8	1.8-4.1	0.000	4.7	2.9-7.7	0.000
Has less than 2 close friends	1.0	0.6-1.8	ns	1.2	0.7-1.9	ns	1.8	1.1-3.1	0.026
Intraclass correlation (min-max)	0.00-0.10			0.01-0.05			0.12-0.21		
Sample size (min-max)	537-595			482-531			588-637		

Odds ratios are adjusted for age only for all the associations appearing in this table.

Age-adjusted associations between risk behaviors and explanatory variables among girls are displayed in Table 3. Considering attempting suicide and truancy were characteristics significantly associated with all three risk behaviors. Insomnia was significantly associated with smoking and cannabis use. Girls who felt sad, whose parents rarely checked their homework, understood their problems, or knew their whereabouts were more likely to report alcohol use. Having parents who rarely check homework was associated with cannabis use in girls.

**Table 3:** Age-adjusted associations between risk behaviors and psychosocial characteristics among girls

	Smoking			Alcohol use			Cannabis use		
	
Variable	OR	95% CI	*P*	OR	95% CI	*P*	OR	95% CI	*P*
Felt sad or hopeless ≥ 2 weeks in a row in past 12 months	1.4	0.8-2.4	ns	1.9	1.3-2.8	0.001	1.8	0.8-4.2	ns
Often felt lonely in past 12 months	1.7	1.0-3.2	0.065	1.4	0.8-2.3	ns	0.6	0.2-2.3	ns
Was so worried about something that could not sleep at night in past 12 months	2.4	1.3-4.5	0.005	1.3	0.8-2.3	ns	2.6	1.0-6.9	0.049
Seriously considered attempting suicide in past 12 months	3.4	2.0-5.8	0.000	2.5	1.5-4.0	0.000	2.8	1.2-6.8	0.018
Parents rarely/never check homework	1.5	0.9-2.6	ns	2.1	1.4-3.1	0.000	2.6	1.1-6.3	0.029
Parents rarely/never understand problems	1.0	0.6-1.7	ns	1.5	1.1-2.2	0.026	1.7	0.7-4.0	ns
Parents rarely/never know what child is doing	1.6	0.9-2.7	0.080	1.7	1.1-2.5	0.009	2.0	0.8-5.1	ns
Pocket money ≥ 25 rupees per day	1.7	1.0-3.0	0.061	1.0	0.6-1.5	ns	1.6	0.6-4.5	ns
School absence without permission ≥ 1 day in past 30 days	5.7	3.3-9.9	0.000	2.7	1.7-4.2	0.000	10.5	3.9-28.5	0.000
Has less than 2 close friends	0.9	0.5-1.7	ns	1.5	1.0-2.3	0.068	0.6	0.2-2.0	ns
Intraclass correlation (min-max)	0.00-0.02			0.08-0.14			0.21-0.25		
Sample size (min-max)	662-704			597-634			679-721		

Odds ratios are adjusted for age only for all associations appearing in this table.

Table 4 displays multivariate associations between risk behaviors and explanatory variables in boys and in girls. Age was significantly associated with smoking and alcohol use among both boys and girls. Among boys, seriously considering attempting suicide and pocket money were factors positively associated with smoking. Parents rarely/never knowing what their children are doing and truancy were factors associated with all three risk behaviors. In addition, insomnia was associated with cannabis use was. Among girls, seriously considering attempting suicide was significantly associated with smoking and with drinking. Pocket money was associated with smoking, whereas having parents rarely/never checking their children's homework and feeling sad were factors associated with drinking. Similar to boys, truancy was strongly associated with all three risk behaviors (OR = 6.1, p < 0.001 for smoking, OR = 2.6, p = 0.001 for alcohol use; OR = 33.2, p < 0.001 for cannabis use).

**Table 4:** Stepwise multivariate association between risk behaviors and psychosocial characteristics in boys and in girls

	Boys						Girls					
	
	Smoking		Alcohol use		Cannabis use		Smoking		Alcohol use		Cannabis use	
	
Variable	OR	*P*	OR	*P*	OR	*P*	OR	*P*	OR	*P*	OR	*P*
Age (year)	1.2	0.030	1.2	0.003	1.2	ns	1.4	0.003	1.4	0.000	0.8	ns
Felt sad or hopeless ≥ 2 weeks in a row in past 12 months	-		-		-		-		1.6	0.023	-	
Often felt lonely in past 12 months	-		-		-		-		-		-	
Was so worried about something that could not sleep at night in past 12 months	-		-		3.6	0.001	-		-		-	
Seriously considered attempting suicide in past 12 months	3.0	0.000	-		2.7	0.002	2.5	0.006	1.8	0.051	-	
Parents rarely/never understand problems	-		-		-		-		-		-	
Parents rarely/never check homework	-		-		-		-		2.3	0.000	-	
Parents rarely/never know what child is doing	1.8	0.016	1.5	0.046	1.9	0.020	-		-		-	
Pocket money ≥ 25 rupees per day	1.8	0.025	-		-		2.6	0.006	-		-	
School absence without permission ≥ 1 day in past 30 days	2.9	0.000	2.3	0.000	2.5	0.001	6.1	0.000	2.6	0.000	33.2	0.000
Intraclass correlation	0.00		0.00		0.00		0.00		0.08		0.26	
Sample size	479		429		515		607		551		624	

*The initial model for each risk behavior included all explanatory variables but only associations that remained significant in multivariate analysis were retained in the final model, except for age which was retained in all models even when not statistically significant.

While confidence intervals were (expectedly) slightly larger when adjusting for a classroom clustering effect, the odds ratios in Table 2 Table 3 and Table 4 were virtually identical in analyses done without classroom clustering adjustment. The intraclass correlations were in the range of 0.00-0.10 for smoking, 0.01-0.14 for alcohol use, and 0.12-0.35 for cannabis use. These results thus suggest a trend towards a larger classroom effect for cannabis use.

## Discussion

We found that substance use (cigarette smoking, alcohol drinking and cannabis use) was associated with age and with several psychosocial (explanatory) characteristics at the individual and family levels. While the prevalence of these risk behaviors tended to differ between boys and girls, predictors of these behaviors did, overall, not markedly differ by gender. At the individual level, considering attempting suicide and truancy were characteristics associated with all three risk behaviors among both boys and girls. At the family level, we found that poor parental monitoring (e.g. having parents who did not check homework regularly or did not know the whereabouts of their children) increased the likelihood of these risk behaviors. These findings suggest that addressing these psychosocial characteristics may extend benefit to the prevention of several risk behaviors.

Consistent with previous reports, the prevalence of the substance use was higher among boys than girls [19,20,23,26,36] and increased with age [19,23]. The gender difference may be partly attributed to socio-cultural norms that tend to stigmatize girls who engage in substance use [19] and/or to larger innate risk taking by males than females [37]. Of note, the protective effect of being a female against engaging in substance use has been documented to be stronger in the African region than in the United States [19], a finding which may be attributed to socio-cultural differences between different regions.

Psychological characteristics such as sadness, loneliness, insomnia due to worrying, and suicidal ideation were associated with substance use. The association with suicidal ideation was particularly strong. Previous studies, including prospective cohorts, have suggested that teenagers who experience depressive symptoms are at increased risk for engaging in substance use during adolescence [4,15] and subsequently in adulthood [38]. It has been suggested that these individuals may try to cope with stress and relieve depression by engaging in substance use [15,38].

We found that all three risk behaviors were associated with poor parenting practices. Our findings are consistent with several studies linking substance use and poor family management [11,12,22,39,40]. Among boys, "parents knowing what their child is doing at leisure time" was the most powerful protective characteristic among the three considered parenting practices variables, consistent with results in previous studies [12]. Among girls, "parents not checking their children's homework" was also associated with substance use. In contrast to previous studies [10,11], we did not find a consistent association between substance use and "parental understanding of adolescents problems". Overall, while parental support and good parent-child communication seem to protect children against substance use, parental monitoring (i.e., parents knowing the whereabouts of their child and checking their child's homework) seemed to be the strongest protective characteristic against these risk behaviors. Given that it has been suggested that programs aimed at improving parent-child communication and parental monitoring are effective in changing adolescent behaviors [41], our findings reinforce the idea that parents (particularly those of "high risk" adolescents) might benefit from participating in such programs.

We also found that the substance use was associated with pocket money, truancy, and having less than two friends. In our study, pocket money was the closest available indicator of socioeconomic status. In line with previous studies, we found that adolescents who had more pocket money than their peers tended to engage more often in the considered risk behaviors. This may relate to increased purchasing power [25], self efficacy, independence, and means to interact socially. Truancy (as measured by missing school without permission) was strongly associated with all three risk behaviors. This association was particularly strong among girls regarding cannabis use (OR = 33, 95% CI: 6.9-160.6), although this finding is based on few cases. While truancy may merely be a marker of other personal risk behaviors and/or underlie an unstable family environment, this characteristic may also reflect increased opportunities to engage in substance use during the unsupervised time out of school [21]. We previously found, in another study among children in Seychelles, that students who were absent from school during a school survey engaged more often in substance use than those present [42].

Having less than two close friends generally increased the likelihood of substance use. In other studies, the association between popularity among peers and substance use in adolescents has been inconsistent. While some reports show that 'popular' teenagers tend to have higher rates of risk-taking behaviors [16] (possibly because a larger social network can increase the opportunities for engaging in risk behaviors), other studies suggest that being popular actually protects against adopting risk behaviors [34]. These findings seem consistent with our observation that loneliness increases substance use, suggesting that social isolation may be a source of stress and boredom, and can lead to increased risk behaviors [24]. These mixed findings underlie the need to consider interactions between different factors, such as the socio-economic status of the students, school settings (e.g. access to substances), cultural background, etc.

Our study has a few limitations. All data were reported by the adolescents and may reflect subjective perception of substance use rather than the actual situation. Our lenient definitions of substance use (i.e., fairly low levels of the considered risk behaviors) may reflect experimentation more than sustained substance use in some cases, and may be regarded as a normal aspect of adolescent development in some instances [6-8]. Moreover, the true prevalence of substance use among adolescents is likely underestimated since it has been shown that students missing school tend to have a higher prevalence of risk behaviors than students not missing school (at the time of a survey) [42]. However, it must be emphasized that the observed associations do not imply causation due to the cross-sectional nature of this study. Multicollinearity can be an issue in multivariate analysis since several explanatory variables tend to express the same dimensions and considering that it is statistically difficult to distinguish the effects on the outcomes of different explanatory variables which are correlated with each other This may be the case for variables such as "parents rarely/never check homework", "parents rarely/never understand problems" and "parents rarely/never know what child is doing". Thus, the fact that a given variable is not retained in our multivariate models in Table 4 does not necessarily imply that this variable has effect on the outcome, but it might be that it is highly correlated with one or more of the other variables retained in the model. Of note, much of the interpretation of our data in this paper, which is descriptive in nature, is based on models that are only adjusted for age (Table 2 and Table 3) and in which multicollinearity does not arise. Finally, the use of scores that assemble questions of similar domains might provide useful insight for investigating the associations between explanatory variables and health behaviors. However, no score has been proposed or validated yet within the standard methodology of GSHS. On the other hand, our study is one of few studies that have examined several risk behaviors simultaneously among adolescents in the sub-Saharan African region.

## Conclusions

We found that substance use (cigarette smoking, alcohol drinking, and cannabis use) in adolescents is associated with several shared psychosocial characteristics. This reemphasizes the need to address the prevention of substance use through comprehensive interventions, as opposed to vertical programs on particular substances. We identified risk factors and protective factors at both the individual and family levels. Correspondingly, health promotion programs should aim at addressing a broad range of factors at these different levels. For example, reinforcing parental support and monitoring has been shown to reduce the prevalence of adolescent substance use [41]. Finally, the prevalence of substance use differed between boys and girls and the strength of the associations with the considered predictors varied according to gender in some instances, which may relate to socio-cultural factors. This underlies that gender differences should be carefully evaluated when formulating prevention interventions.

## Competing interests

The authors declare that they have no competing interests.

## Authors' contributions

HA led the write up of the manuscript and the analysis of the data; BV organized and supervised the conduct of the survey and reviewed the manuscript; VR assisted in data analysis and participated in the write up of the manuscript; FP reviewed the manuscript; PB participated in the study design, data analysis, and in the write up of the manuscript. All authors read and approved the final manuscript.

## Pre-publication history

The pre-publication history for this paper can be accessed here:

http://www.biomedcentral.com/1471-2431/11/85/prepub
